# Marker-Less Video Analysis of Infant Movements for Early Identification of Neurodevelopmental Disorders

**DOI:** 10.3390/diagnostics15020136

**Published:** 2025-01-08

**Authors:** Roberta Bruschetta, Angela Caruso, Martina Micai, Simona Campisi, Gennaro Tartarisco, Giovanni Pioggia, Maria Luisa Scattoni

**Affiliations:** 1 Italian National Research Council, Institute for Biomedical Research and Innovation, Via Leanza, Istituto Marino, 98164 Messina, Italy; roberta.bruschetta@irib.cnr.it (R.B.); simona.campisi@irib.cnr.it (S.C.); giovanni.pioggia@cnr.it (G.P.); 2 Research Coordination and Support Service, Istituto Superiore di Sanità, Viale Regina Elena 299, 00161 Rome, Italy; angela.caruso@iss.it (A.C.); martina.micai@iss.it (M.M.); marialuisa.scattoni@iss.it (M.L.S.)

**Keywords:** artificial intelligence, analysis of infants’ movements, neurodevelopmental disorders, automatic motion tracking, deep learning, early identification of neurodevelopmental disorders

## Abstract

**Background/Objectives:** The early identification of neurodevelopmental disorders (NDDs) in infants is crucial for effective intervention and improved long-term outcomes. Recent evidence indicates a correlation between deficits in spontaneous movements in newborns and the likelihood of developing NDDs later in life. This study aims to address this aspect by employing a marker-less Artificial Intelligence (AI) approach for the automatic assessment of infants’ movements from single-camera video recordings. **Methods:** A total of 74 high-risk infants were selected from the Italian Network for Early Detection of Autism Spectrum Disorders (NIDA) database and closely observed at five different time points, ranging from 10 days to 24 weeks of age. Automatic motion tracking was performed using deep learning to capture infants’ body landmarks and extract a set of kinematic parameters. **Results:** Our findings revealed significant differences between infants later diagnosed with NDD and typically developing (TD) infants in three lower limb features at 10 days old: ‘Median Velocity’, ‘Area differing from moving average’, and ‘Periodicity’. Using a Support Vector Machine (SVM), we achieved an accuracy rate of approximately 85%, a sensitivity of 64%, and a specificity of 100%. We also observed that the disparities in lower limb movements diminished over time points. Furthermore, the tracking accuracy was assessed through a comparative analysis with a validated semi-automatic algorithm (Movidea), obtaining a Pearson correlation (R) of 93.96% (88.61–96.60%) and a root mean square error (RMSE) of 9.52 pixels (7.29–12.37). **Conclusions:** This research highlights the potential of AI movement analysis for the early detection of NDDs, providing valuable insights into the motor development of infants at risk.

## 1. Introduction

Neurodevelopmental disorders (NDDs) are a heterogeneous group of conditions that typically manifest during early childhood and involve impairments in the growth and development of the nervous system. These conditions can affect various aspects of a person’s functioning, including behavior, cognition, communication, and social interaction. They include a wide range of conditions, such as autism spectrum disorder (ASD), attention deficit hyperactivity disorder (ADHD), intellectual disability, specific learning disorders, and communication disorders, among others [1]. Timely diagnosis of NDD plays a crucial role in facilitating effective intervention and support for children and their families, having a profound impact on their long-term developmental outcomes [2].

Recent research has unveiled a deepening comprehension of the intricate interplay among developmental systems during infancy, highlighting how advancements in one area can trigger cascading effects, influencing an infant’s progress in other domains. Referred to as the “developmental cascades” perspective, this viewpoint has been the subject of scrutiny, with notable reviews [3,4,5]. The “developmental cascades” perspective holds significant implications for understanding the consequences of subtle early differences and delays in child development. It suggests that even minor disruptions in early behaviors, especially in seemingly unrelated domains, such as motor skills and language development, can set in motion a sequence of direct and indirect consequences [6]. In essence, slight variations or delays in early motor behaviors can set off a series of events that impact the development of language and communication skills and lead to developmental divergences over time. This point of view highlights the intricate and interlinked nature of child development, emphasizing how important early intervention and support are in addressing even minor developmental differences. Furthermore, extensive research indicates that the early phases of motor development can be a pivotal lens for assessing the broader neurodevelopmental trajectory in children. Delays in achieving motor milestones may suggest that the child’s development is not progressing as expected and may be indicative of underlying neurodevelopmental concerns [7]. Therefore, assessing motor skills in the first weeks of life takes on significant importance.

Clinical assessments of motor performance in infants within the scope of neurodevelopment evaluation typically employ standardized tools and observational methods. These assessments help healthcare professionals in evaluating an infant’s motor development and identifying potential delays or abnormalities. Widely used assessment tools include the General Movements (GMs) Assessment [8,9], the Alberta Infant Motor Scale [10], and the Peabody Developmental Motor Scales [11]. Specifically, the assessment of GMs has emerged as a clinical method to evaluate early motor abilities and has been extensively employed in the context of early neurodevelopment assessment of preterm newborns [12,13,14]. Several studies have highlighted the relevance of GMs in predicting developmental outcomes and the potential for early intervention. However, these methods have some limitations, including subjective assessment, time-consuming procedures, susceptibility to environmental factors, and the need for specialized expertise. Additionally, the capacity for continuous monitoring and predictive precision stands as potential challenges within clinical assessments. In response to these challenges, researchers are actively exploring technology-driven solutions to address some of these limitations and provide more objective and continuous insights into infants’ motor development.

Many studies have employed wearable sensors, such as the work conducted by Smith et al. [15], who investigated sample entropy from tri-axial accelerometer and gyroscope data as a metric for assessing the variability of spontaneous leg movements in infants, with the potential to serve as an indicator of neuromotor control development. Another line of research has focused on applying computer vision and machine learning techniques to develop innovative tools for directly assessing movements from video recordings of children. A scoping review of studies exploiting computer vision and machine learning is reported in [16]. Among these, Caruso et al. [17] presented Movidea, a novel semi-automatic software designed to support movement tracking and GMs Assessment in high-risk infants for ASD from video recordings. Another contribution comes from Tsuji et al. [18], who introduced a marker-less movement measurement method designed to evaluate 25 indices linked to GMs in infants with the aim of enabling early disability diagnosis. Video analysis includes background subtraction, frame difference computations, and movement classification and is executed using an artificial neural network characterized by a stochastic structure.

Furthermore, recent studies have explored complementary approaches, focusing on gamified platforms and machine learning to analyze behavioral features in older children for diagnosing neurodevelopmental disorders such as ASD and ADHD. For example, a study by [19] developed a system integrating crowdsourcing and machine learning to extract multimodal features, highlighting the scalability and adaptability of such approaches in clinical and home environments. While these methodologies address older age groups, they underscore the potential of computational tools to enhance neurodevelopmental assessments and inform real-time decision-making.

Notably, the introduction of AI stands out, presenting significant potential for the early and objective evaluation of infants’ motor skills. This approach not only enhances the efficiency of the assessment process but also introduces the prospect of continuous monitoring and in-depth analysis of motor development. A key advantage is that these methods obviate the need for sensors, affording infants the freedom to move naturally during the assessment [20]. Several AI-driven solutions have been proposed and employed for automated movement tracking. In a recent study by Shin et al. [21], automatic movement tracking based on AlphaPose was applied to high-risk preterm infants in the context of cerebral palsy. The research involved kinematic analysis of joint angles and angular velocities using sample entropy and Pearson correlation. The aim was to examine inter-limb synchronization and movement complexity of both upper and lower extremities during spontaneous movements for assessing developmental outcomes. Reich et al. [22], in a different study, used OpenPose to identify age-specific movement patterns known as “fidgety movements (FMs)” in TD infants. The infants’ complete body poses were extracted from videos, resulting in a 25-point skeleton, which served as the input data for a shallow multilayer neural network (SMNN). DeepLabCut was recently employed in another study by Moro et al. [23] to analyze spontaneous movements in preterm infants and detect abnormal motion patterns indicative of neurological disorders. Their paper introduces a computer-aided pipeline for processing 2D video recordings to characterize and classify infants’ motion. The pipeline involves detecting key body points to extract quantitative motion parameters for subsequent classification, distinguishing between normal and abnormal motion patterns. In a longitudinal study, Doi et al. [24] employed machine learning models to explore the connection between spontaneous bodily movements in 4-month-old infants and the development of ASD-like behaviors at 18 months by extracting 26 movement features through interframe difference images from video recordings. Subsequently, in another recent study [25], they applied the same marker-less approach to investigate the association between spontaneous movements in infants at approximately two days of age, particularly during sleep, and their risk of developing ASD at 18 months. The findings revealed that a higher risk of ASD is correlated with specific motor alteration patterns during sleep and awake states, pertaining to the rhythm and movement frequency of both the lower and upper body. Beyond these works, neural networks have been widely employed in various studies for the automated tasks of pose estimation and movement classification [26,27].

In the context of an escalating interest within the scientific community, this study introduces an innovative application of AI to automatically analyze spontaneous movements in newborns identified as high-risk for NDD. Employing video footage acquired at five discrete time points spanning from 10 days to 24 weeks postnatally, our objective was to identify and underscore novel early motor markers indicative of potential developmental delays. The employed methodology is marker-less, eliminating the need for direct operator intervention and thereby emphasizing its efficiency and non-intrusiveness. This research contributes valuable insights to the understanding of early neurodevelopmental indicators and holds implications for non-invasive monitoring in high-risk populations.

## 2. Materials and Methods

### 2.1. Participants

For this study, 74 high-risk (HR) infants (11F/9M) were recruited from the Italian Network for Early Detection of Autism Spectrum Disorders, also known as the NIDA network [28]. The NIDA network is the largest Italian cohort of infants at risk for NDDs and includes siblings of children diagnosed with ASD, preterm newborns, and small-for-gestational-age newborns, as well as low-risk (LR) infants, who serve as a comparison group. This network was established to detect early signs of NDD, including ASD, through a multidisciplinary approach that integrates motor and cry pattern analysis with comprehensive clinical assessments. For each participant, five videos capturing the infant in a state of natural and unrestricted movements at five specific time points corresponding to 10 days and 6, 12, 18, and 24 weeks of age were recorded and analyzed. These time points were chosen to capture crucial moments in motor development from birth onward. Furthermore, at 36 months of age, a comprehensive clinical/diagnostic assessment of the infants and toddlers using standardized tools/tests and parental structured interviews was conducted to confirm the presence/absence of NDD. Within this cohort, 5 infants dropped out of the study, 28 received a diagnosis of NDD, and 33 were assessed as children with typical development (TD). Additionally, 8 infants are still pending evaluation since they have not yet reached the appropriate age for a stable diagnosis. Videos of no-label or drop-out infants were used exclusively for validating our deep learning-based automatic tracking approach and not for the subsequent analysis steps aimed at identifying predictive variables for NDD. The dataset (summarized in Table 1) lacks complete sets of five videos for each participant, as not all individuals could be recorded at every designated time point. Furthermore, in addition to the videos reported in the dataset and recorded as described in the following paragraph, we used four additional amateur-recorded videos to test our tracking algorithm under completely unstructured conditions. Further details about the dataset can be found in Supplemental Table S1.

The study was approved by the ethics committee of the National Institute of Health (Istituto Superiore di Sanità, ISS) (Approval Number: Pre 469/2016). All the families that voluntarily participated in the study signed a written informed consent, and all methods were performed in accordance with the relevant guidelines and regulations. Informed consent for publication of identifying information/images in an online open-access publication was also obtained from the parents of the subjects whose images appear in this manuscript.

### 2.2. Data Collection & Video Editing

Infants were recorded in a home setting, lying on a green blanket supplied by the NIDA network. The camera was positioned at a distance of 50 cm above the infant’s chest. Each recording lasted for a minimum of 5 min, aiming to capture spontaneous movements of the infant’s entire body. Recorded videos were reviewed, revealing that in most cases, segments of high quality (i.e., without external disturbances such as operator intervention to soothe the infant) did not exceed 3 min in duration. Consequently, the decision to retain a 3 min video segment representing the highest-quality portion for each recording was made. To ensure consistency in the dataset, one author manually edited the videos to meet the following specific criteria: a duration of three minutes and the infant in a supine position, displaying a state of well-being and spontaneous motor activity, and without any episodes of crying. In cases where the videos contained more than 3 min of high-quality footage, we opted to analyze the initial 3 min of high-quality content. Any video frames that exhibited operator or parental interferences, as well as accidental camera movements, were excluded from the subsequent analysis. All the analysis presented in the following sections was performed using Python 3 [29] and Matlab R2022b [30].

### 2.3. Tracking Procedure

**Figure 1:**
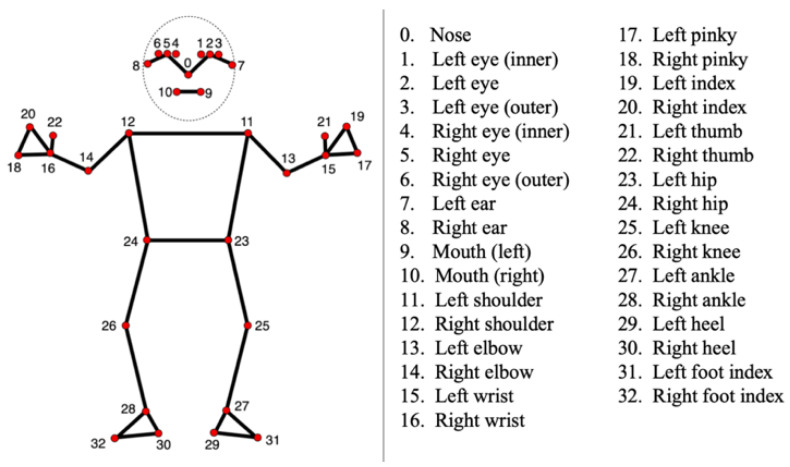
List of the 33 body landmark locations tracked using the Mediapipe Pose solution [34].

The trajectories of infants’ body landmarks during their spontaneous movements were automatically extracted from all the collected videos using a customized application of the Mediapipe Pose Landmark Detection solution [31]. MediaPipe is an open-source framework developed by Google for creating AI and machine learning pipelines, providing a suite of libraries and tools for multimedia processing across multiple platforms. The MediaPipe Pose Landmarker, a specific component of this framework, facilitates the detection of human body landmarks within images or videos, allowing for the identification of key body points, posture analysis, and movement categorization. The architecture, based on BlazePose [32], a specifically modified version of the MobileNet [33] convolutional neural network (CNN), outputs 33 body pose landmarks, expressed in both image coordinates (pixel values) and 3-dimensional world coordinates. These landmarks correspond to significant body components, including key joints and extremities, specifically hands and feet. The comprehensive list of landmarks is provided in Figure 1. For each reference point, we stored an Nx2 matrix containing the x and y coordinates in the image for each of the N frames in the respective video.

We selected the MediaPipe Pose Landmark Detection as the body pose estimation solution for our study due to several advantages. Notably, it has undergone extensive training on large datasets and fine-tuning to ensure precise landmark detection. Additionally, it offers real-time pose estimation with minimal latency. Furthermore, the CNN architecture is easily customizable to meet specific project requirements.

**Figure 2:**
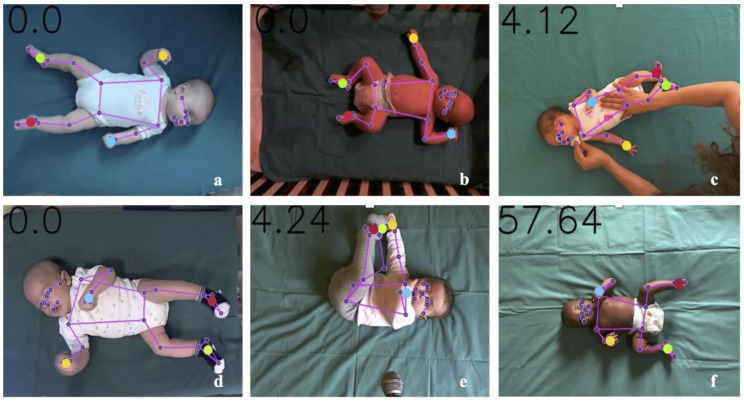
Examples of videos with low resolution (**a**), poor lighting (**b**), hand operator intrusion in the frame (**c**), and children wearing socks and clothes that covered their limbs (**d**,**e**) or had different skin tones (**f**).

This AI approach in our study has demonstrated remarkable robustness also in conditions such as low resolution (Figure 2a), inadequate lighting (Figure 2b), inadvertent operator intrusion into the frame (Figure 2c), infants wearing socks or clothing that covered their limbs (Figure 2d,e), or variations in skin tones (Figure 2f). In all these scenarios, motion tracking was performed accurately, as further confirmed by the model validation described in the next sections.

### 2.4. Signal Processing

**Figure 3:**
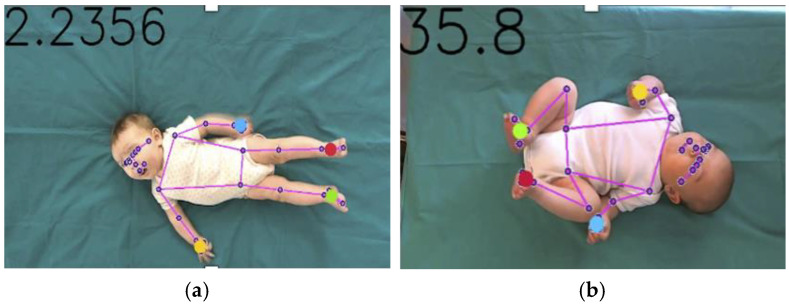
Examples of two frames with the detected reference points and the corresponding skeleton overlaid. The hands and feet centroids are indicated by larger dots. Figure (**a**) shows the overlaid skeleton when the limbs are fully extended, whereas Figure (**b**) illustrates the case where the limbs are bent.

Using the outlined methodology, we identified the x and y coordinates of 33 body landmarks for each frame of each video in our dataset. The AI model ensures consistency across diverse frame sizes by normalizing coordinate values within the range of 0 to 1. The output for each processed video was a file containing the x and y coordinates of each landmark across every frame in which the model successfully performed body pose estimation. For subsequent analyses aimed at identifying early motor predictors of NDDs, we specifically focused on infants’ limbs. The centroid coordinates of each end-effector were computed for each frame, mediating the x and y coordinates of the wrist, pinky, and index for the hands and of the ankle, heel, and foot index for the feet (Figure 3). Each signal was then preprocessed by applying a zero-phase moving average filter with a selected window size of 5 samples in order to smooth noisy fluctuations.

### 2.5. Model Validation: Comparison with Movidea

To evaluate the performance of our automatic tracking approach, we conducted a comparative analysis between the trajectories derived from our procedure, described in the previous sections, and those obtained using Movidea [35], a validated software package purposefully designed for the semi-automatic extraction of kinematic features in assessing infants’ motor skills. Movidea is software that facilitates the tracking of infants’ end-effectors during the free movement of each limb from single-camera video footage. However, it requires the presence of an operator for the selection of the infant’s reference measures, specifically the head length and the symmetry line of the body. The operator is also responsible for selecting the central point of each limb to initialize the tracking algorithm, and he must oversee the entire process, intervening to reset the points in the event of errors and skip frames in cases where a limb is not visible.

During the validation procedure, in addition to the videos listed in Table 1 acquired through the method described in Section 2.2, we also tested four additional amateur videos. These videos were recorded under conditions different from the structured procedure established for the study. To assess the similarity between our tracking results and those from the operator-dependent software Movidea, we computed the Pearson’s correlation coefficient (R), the *p*-value testing the hypothesis of no relationship between the two signals, and the root mean square error (RMSE). For each signal, similarity measures were computed within 10 s windows and then averaged across all these windows. Subsequently, we checked the normality of the data distribution for each measure using the Shapiro–Wilk statistical test. Upon identifying non-normal distributions (*p* < 0.001), we expressed the aggregated results across all the signals using the median value (Q1–Q3).

### 2.6. Feature Extraction

To characterize the spontaneous movements of infants in terms of synchronicity, smoothness, repetitive movements, and symmetry, we used the trajectories of each end-effector’s centroid extracted from videos of NDD and TD children at each time point to compute the following features and kinematic parameters:

- Velocity and Acceleration:

The velocity of each limb was calculated as the Euclidean distance between the location of the corresponding reference point in every two subsequent frames. The obtained signal was then multiplied by the frame rate to normalize any difference due to videos captured using different devices. To reduce the fast oscillations in the velocity profiles, a third-order low-pass Butterworth filter with a cutoff frequency equal to 95% of the Nyquist frequency was then applied. Analogously, the acceleration of each limb was calculated as the difference between two consecutive velocity samples [35]. Descriptive statistics, including mean, median, standard deviation, variance, mode, skewness, kurtosis, maximum, and minimum, were then computed for both the velocity and acceleration of each limb. Furthermore, the features obtained for the right and left hands, as well as for the right and left feet, were averaged to aggregate the kinematics of the upper body and lower body, respectively.

- Cross-correlation (CC):

The calculation of the zero-lag cross-correlation between the velocity of each pair of limbs was performed by applying the following equation [36]:

(1) $${CC}_{v_{1}v_{2}}=\;\frac{\sigma_{v_{1}v_{2}}}{\sqrt{\sigma_{v_{1}}^{2}{\cdot \sigma }_{v_{2}}^{2}}}$$

where ${CC}_{v_{1}v_{2}}$ is the cross-correlation between the velocity $v_{1}\;$ and the velocity $v_{2}$, $\sigma_{v_{1}v_{2}}$ is the covariance of $v_{1}$ and $v_{2}$, $\sigma_{v_{1}\;}^{2}$ is the variance of $v_{1}$, and $\sigma_{v_{2}}^{2}$ is the variance of $v_{2}$.

Cross-correlation (*CC*) serves as a metric to evaluate the synchronization of limb movements, fundamental information for describing the distinct motor patterns exhibited by infants. Consequently, it holds significant importance in the early assessment of NDD [37].

- Area differing from moving average (Ama):

The smoothness is another crucial parameter for the assessment of infants’ proper development, as typical GMs are characterized by continuous, flowing patterns without jerky or abrupt transitions. To quantify this aspect, we computed the moving average for both the x and y components of each limb’s trajectory across the entire recording using a 2 s window size [37], as described by the following equation:

(2) $$x_{i}=\frac{1}{k}\sum_{j=i-\frac{k}{2}}^{i+\frac{k}{2}}x_{j}$$

where $x_{i}$ is the moving average computed at the *i*-th frame, *k* is the window’s size, and $x_{j}$ is the point position in the *j*-th frame.

Successively, for every sample in the trajectory, we calculated the deviation from the moving average by subtracting the trajectory value from the corresponding moving average value, as shown in the following equation:

(3) $$A_{max}=\sum_{i=\frac{k}{2}}^{l-\frac{k}{2}}\left|x_{i}-{\underline{x}}_{i}\right|$$

where $A_{max}$ is the area differing from the moving average of the *x* component and *l* is the total number of frames of the recording.

Finally, the total area differing from moving average (Ama) was computed for both the lower and upper limbs. This was accomplished by summing the areas that deviated from the moving average for the two components of the feet and the two components of the hands, respectively.

- Periodicity:

Periodicity, as defined by Meinecke L. et al. [37], is a parameter designed to assess the presence of repetitive movements in limb motion. To measure the periodicity of infants’ spontaneous movements, we computed a high-order moving average for both the x and y components of each limb’s trajectory across the entire recording, and we segmented the recordings using a 1000-sample window size. This window length was chosen to capture the overall movement trend without closely tracking the trajectory, allowing us to detect fast movements with high amplitude [37]. Within each window, we computed the mean trajectory for each limb’s movement component, and we then identified the points where the trajectory intersected with the mean. Subsequently, we calculated the mean distance (d) and standard deviation ($\sigma_{d}$) between consecutive intersections. Finally, the periodicity (*P*) was determined by combining the aforementioned parameters as follows:

(4) $$P=\frac{1}{d+\sigma_{d}}$$

- Maximum displacement along the *x* and *y* axes:

This was calculated for each limb by subtracting the minimum coordinate from the maximum coordinate occupied by the reference point during the recording. This measurement was employed to quantify motion amplitude, another important characteristic given that typical GMs exhibit high range and extent.

- The smallest and largest eigenvalues of the 95% error ellipse between the x and y components of the body centroid trajectory:

A covariance error ellipse in two dimensions is a graphical representation of the spread or dispersion of a bivariate distribution. It is centered on the mean value of the two variables, and its shape and orientation depend on the covariance between them. The major and minor axes of the ellipse correspond to the directions of greatest variation in the data (eigenvectors), as determined by the covariance matrix. The orientation of the ellipse indicates the correlation between the variables. A longer major axis implies greater variability in that direction and vice versa.

The eigenvalues of the covariance matrix represent the variance of the data along the eigenvectors. In the case of correlated data, the eigenvectors indicate the direction of the largest spread, while the eigenvalues indicate the magnitude of that spread.

Therefore, covariance error ellipses are helpful in understanding the distribution of data points and, within our specific application, can be used to quantify the magnitude of motion [38].

- Percentage of covered space:

For each limb, we computed the ratio between the sum of all the different positions occupied by that limb and the total number of pixels in the frame to quantify the area of movement.

- Mean Pearson Correlation Coefficient [39]:

This was computed between the x and y components of the left and right hands and between the left and right feet trajectories to also measure the symmetry between the two sides.

- The difference between the mean velocity of upper body and the mean velocity of lower body:

This has been calculated with the aim of comparing the amount of movement between the upper and lower parts of the body according to the following formula:

(5) $$v_{diff}=v_{lefthand}+v_{righthand}-v_{leftfoot}+v_{rightfoot}$$

where $v_{diff}\;$ is the difference between the mean velocity of upper body and the lower body, $v_{lefthand}\;$ is the mean velocity of left hand, $v_{righthand}\;$ is the mean velocity of right hand, $v_{leftfoot}\;$ is the mean velocity of left foot, and $v_{rightfoot}\;$ is the mean velocity of right foot.

Features were normalized by the infant’s bust size to remove differences due to children’s size. Infants’ bust measure was computed by calculating the Euclidean distance between the midpoint of the shoulders and the midpoint of the hips for each frame and subsequently extracting the median of these values.

### 2.7. Feature Selection

Applying the feature extraction procedure detailed in the preceding section, we derived a comprehensive set of parameters that characterize the spontaneous movements of each infant in our dataset across both the NDD and TD groups and throughout all time points. These features comprised 45 parameters, encompassing a range of kinematic variables tailored to assess the gross motor skills of infants. Specifically, they included 18 statistical descriptors related to velocity and acceleration for both lower and upper limbs, 6 cross-correlations among limb pairs, 2 parameters capturing the periodicity of upper and lower body movements, and 2 metrics representing deviations from the moving average for the upper and lower limbs. Additionally, our extraction process encompassed 8 features associated with the maximum displacement of each limb along the *x* and *y* axes, 2 eigenvalues of the centroid, 4 indicators measuring the percentage of space occupied by each limb, 2 Pearson correlation coefficients for upper and lower body movements, and 1 feature expressing the difference in velocity between hands and feet. All these variables were examined to identify potential early predictors of clinical outcomes, specifically distinguishing between children diagnosed with NDD and those with typical development. The following procedure was independently executed for each time point after applying min-max scaling normalization, which accounted for differences in measurement units and value ranges of features. As the initial step in reducing the high number of variables, we conducted a correlation analysis using the Pearson method. When variable pairs showed a correlation exceeding 80%, we systematically removed the one displaying lower variability within our sample. Subsequently, we utilized the ANOVA *F*-value between the labels (NDD versus TD) and each of the remaining features to rank them and identify the optimal subset of variables for each time point [40,41]. In the context of feature selection, the one-way ANOVA assesses the significance of a feature in explaining the variance in the data and distinguishing between the NDD and TD groups. Specifically, the *F*-value used in this test quantifies the ratio of the variance between groups to the variance within groups. A high *F*-value indicates that the feature is more relevant for distinguishing between groups, making it a strong candidate for feature selection [42]. The *F*-value is calculated as the ratio of the mean squares between groups (*MSB*) to the mean squares within groups (*MSW*):

(6) $$F=\frac{MSB}{MSW}$$

The variance between the group means (*MSB*) is calculated by dividing for the degrees of freedom for between groups (dfB), equal to *k* − 1, where *k* is the number of groups, the Sum of Squares Between Groups (SSB):

(7) $$SSB=\sum n_{i}{\left(\mu_{i}-\bar{\mu }\right)}^{2}\;$$

where $n_{i}$ is the number of observations in the *i*-th group, $\mu_{i}$ is the mean of the *i*-th group, and $\bar{\mu }$ is the overall mean.

The variance within each group (*MSW*) is instead measured by dividing for the Degrees of freedom for within groups (dfW), equal to N − *k*, where N is the total number of observations, the Sum of Squares Within Groups (SSW):

(8) $${SSW}_{i}=\sum {\left(x_{i}-\mu_{i}\right)}^{2}$$

Here, ${SSW}_{i}$ is the sum of squared differences between individual data points $x_{i}$ and their group mean $\mu_{i}$ for each group *i*. The total SSW is the sum of these ${SSW}_{i}$ values across all groups.

To select the optimal number of features for the subset at each time point, we employed a recursive algorithm. Starting with the subset comprising the two features that obtained the highest-ranking scores, the algorithm computed classification performance at each iteration while sequentially adding features in descending order of their ranking. Ultimately, we chose the optimal subset that achieved the best performance with the fewest features. The selected variables at each time point were then compared between the two groups using the non-parametric unpaired two-sample Wilcoxon statistical test.

### 2.8. Mixed-Effects Model for Selected Features

Given the longitudinal nature of our dataset, with repeated measurements for each subject at different time points, we decided to conduct an additional analysis focused only on the features selected as described in the preceding paragraph to address the potential correlation among repeated measures within the same individual. We used a mixed-effects model, which effectively handles this complex data structure, including missing data [43]. This approach enabled us to thoroughly examine our variables by incorporating fixed effects for Group (TD vs. NDD), Time Point, and the Group–Time Point interaction. Random effects were also included to account for within-subject variability (1|Subject). We used Restricted Maximum Likelihood (REML) as the estimation method. The model applied to analyze each feature was defined as follows:

Selected Feature ~ 1 + Time Point + Group + Time Point: Group + (1|Subject)(9)

When significant results were found, we performed post hoc analyses to interpret them.

### 2.9. Classification Model

The selected and normalized features were employed as input to train a C-Support Vector Classifier (C-SVC) [44] for the early identification of NDD infants at a specific time point. C-SVC is a specific type of Support Vector Machine (SVM) designed to find an optimal decision boundary or hyperplane for effectively separating different classes of data in a dataset, ensuring the maximum margin between the classes while still allowing for some classification errors based on the chosen value of C. C represents a regularization parameter that controls the trade-off between maximizing the margin (the distance between the decision boundary and the nearest data points of each class) and minimizing classification errors. A smaller value of C will result in a larger margin but may allow some misclassification of data points, while a larger value of C will lead to a narrower margin but fewer misclassifications. This algorithm is particularly useful when dealing with non-linearly separable data, as it can map the data into a higher-dimensional space to find a linearly separable hyperplane; moreover, several studies have demonstrated the effectiveness of this classification model, even with small-scale datasets. In our work, we set C = 1, and we chose a radial basis function kernel since data are not linearly separable. Leave-one-out Cross-Validation (LOOCV) [45] was employed to train and test the SVM classifier, considering the limited size of the dataset [44]. The performance was assessed using the following metrics [46]:

- Accuracy:

This metric evaluates the ratio of correct predictions to the total number of instances.

- Precision:

Precision represents the ratio of true positive instances to the total instances classified as positive. It assesses the classifier’s capability to refrain from mislabeling a negative sample as positive. A high precision value indicates a low rate of false positives, suggesting that when the model predicts a positive result, it is likely correct.

- Recall or Sensitivity:

This metric measures the proportion of positive instances that are accurately classified. Recall reflects the classifier’s ability to identify all positive samples.

- Specificity:

Specificity gauges the proportion of true negatives correctly identified by a classification model out of the total number of actual negatives. Essentially, it quantifies the model’s ability to avoid false positives.

- F1 Score:

The F1 score is the harmonic mean of precision and recall. It provides a balanced assessment where the relative contributions of precision and recall are equal.

## 3. Results

### 3.1. Model Validation with Movidea

**Figure 4:**
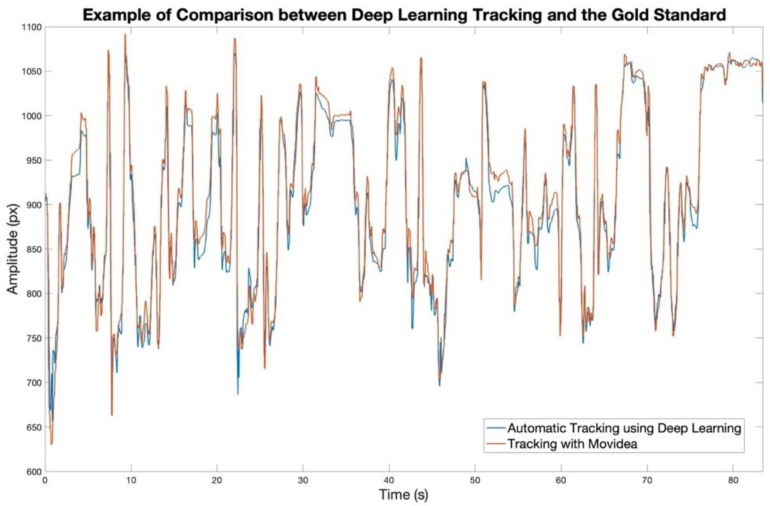
Overlay of a foot trajectory extracted using our deep learning-based automatic approach (blue line) and the same trajectory obtained with the Movidea software used as the gold standard for validating our approach (red line).

The Pearson correlation coefficient between each signal extracted using our automatic tracking approach and its corresponding value obtained from Movidea is 93.96 (88.61–96.60)%. The RMSE was 9.52 (7.29–12.37) pixels. The *p*-value < 0.0001 evidenced a significative relationship between the two signals. An example comparing a trajectory extracted using our deep learning-based automatic approach with the same trajectory obtained from Movidea is provided in Figure 4.

**Figure 5:**
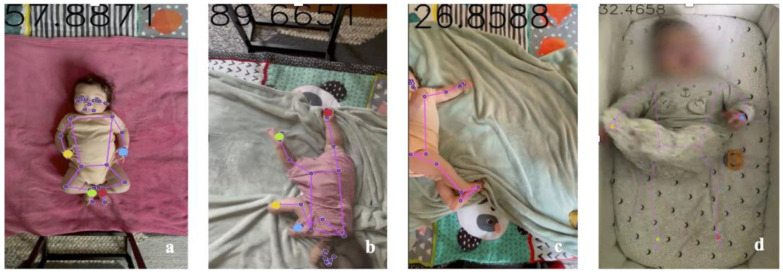
Examples of amateur-recorded videos in unstructured conditions: (**a**) no green background, (**b**) no supine position, (**c**) infant outside the camera’s view, and (**d**) infant clothed in a color similar to the bedsheet.

Testing the four amateur videos, we achieved an R of 95.75% and an RMSE of 8.31 px, even when the background was not green (Figure 5a). Our approach also demonstrated robust tracking capabilities, achieving an R of 91.44% and an RMSE of 10.62 px, even when the child was not in a supine position (Figure 5b). However, for accurate tracking, it was essential that the entire body of the infant remained within the camera’s view, as also evidenced by the evaluation of missing data. The algorithm’s performance when the infant moved outside the frame was R = 42.56% and RMSE = 19.7 px (Figure 5c). Additionally, if the infants were dressed, their clothing needed to have a distinct color from the bedsheet or surface they were lying on. Tests that did not adhere to these constraints resulted in poorer performance, with an R of 64.58% and an RMSE of 11.69 px (Figure 5d).

### 3.2. Feature Selected and Mixed-Effects Model

The most important features identified through the feature selection procedure from the total set of 45 include the ‘Median Velocity of the Feet’, ‘Area differing from moving average’, and ‘Periodicity’, all within the lower-body domain.

**Figure 6:**
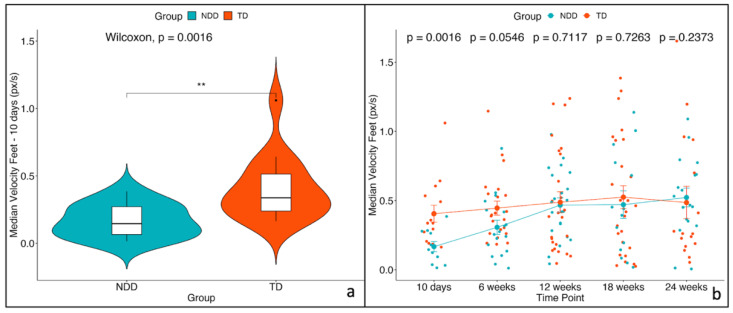
Median Velocity of Feet—(**a**) violin plot for the first time point (10 days) and (**b**) trend across the 5 time points with mean values and the standard errors (SEs) for the two groups: NDD (light blue) and TD (red). The *p*-values related to the comparison between the two groups were computed using the unpaired two-sample Wilcoxon test. * indicates significance levels as follows: * *p* < 0.05, ** *p* < 0.01, *** *p* < 0.001.

**Figure 7:**
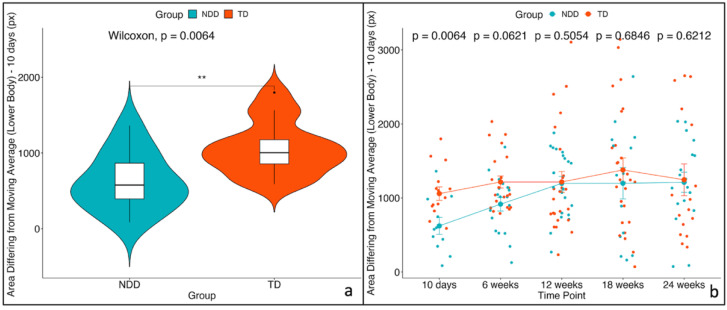
Area differing from moving average of lower body—(**a**) violin plot for the first time point (10 days) and (**b**) trend across the 5 time points with the mean values and the standard errors (SEs) for the two groups: NDD (light blue) and TD (red). The *p*-values related to the comparison between the two groups were computed using the unpaired two-sample Wilcoxon test. * indicates significance levels as follows: * *p* < 0.05, ** *p* < 0.01, *** *p* < 0.001.

**Figure 8:**
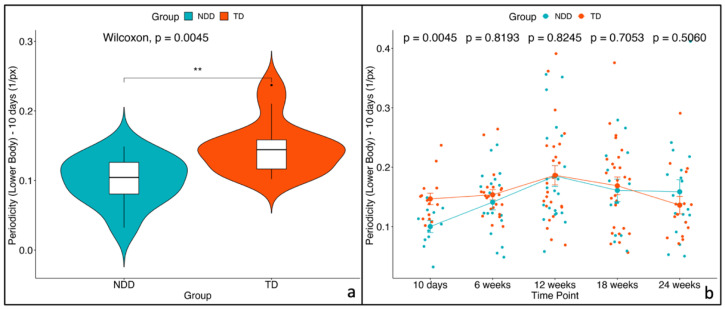
Periodicity of lower body—(**a**) violin plot for the first time point (10 days) and (**b**) trend across the 5 time points with the mean values and the standard errors (SEs) for the two groups: NDD (light blue) and TD (red). The *p*-values related to the comparison between the two groups were computed using the unpaired two-sample Wilcoxon test. * indicates significance levels as follows: * *p* < 0.05, ** *p* < 0.01, *** *p* < 0.001.

A non-parametric unpaired two-sample Wilcoxon statistical test between TD vs. NDD showed that the median values of these three variables were significantly lower in NDD infants than in TD at 10 days. No significant effects were observed for the remaining time points (Figure 6, Figure 7 and Figure 8). Descriptive statistics for these three variables are reported in Table 2.

The mixed-effects model applied across the multiple time points (repeated measures) revealed a significant impact of both time and group (*p* < 0.05) for all three variables. However, the interaction between group and time did not reach statistical significance. Post hoc analyses confirmed that these three variables are significantly different between the two groups only at the initial time point (10 days). The results of post hoc tests are reported in Table 3. To verify that the assumptions were met, we examined the residual plots to confirm the absence of deviations from homoscedasticity or normality.

### 3.3. Classification

**Figure 9:**
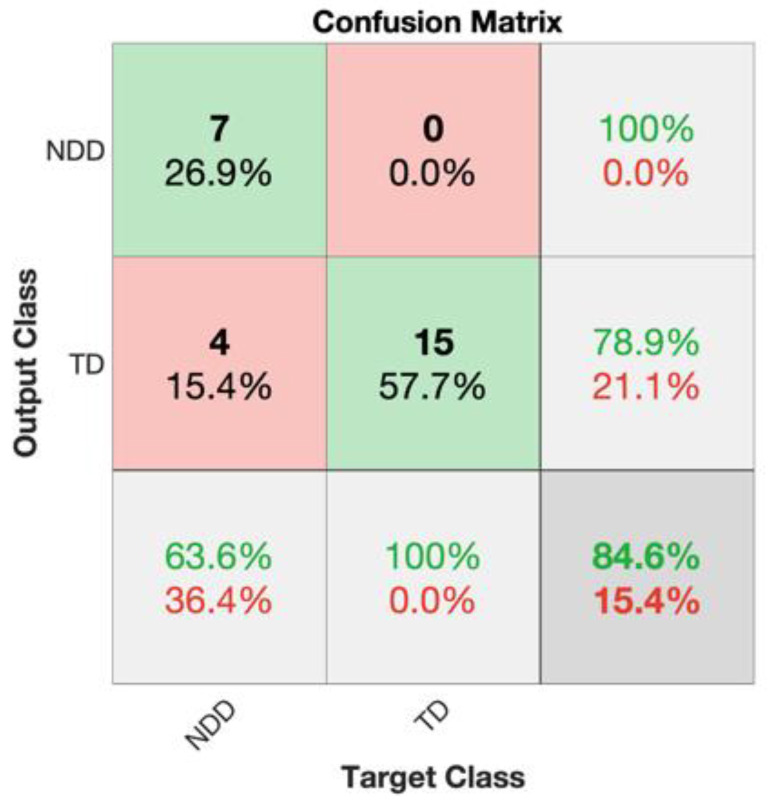
Confusion matrix showing the percentages of subjects accurately and mistakenly associated with each class (children who developed NDD and TD children) for the first time point (10 days). Correctly classified data are shown in green, while misclassified data are shown in red. The rightmost column, denoted as precision, indicates how many infants assigned to a particular group by the classifier truly belong to that class. Similarly, the bottom row of the matrix shows the recall or sensitivity, indicating for each class how many of the total subjects of that class were correctly recognized by the classifier and providing valuable insights into the accuracy and reliability of the classification process. The total accuracy is displayed in the lower right corner.

The combination of the three selected features was used as input for the SVM classifier to distinguish between NDD high-risk infants and TD infants, achieving an accuracy of 84.6%. Details of the metrics are reported in Table 4, and the confusion matrix is shown in Figure 9.

**Figure 10:**
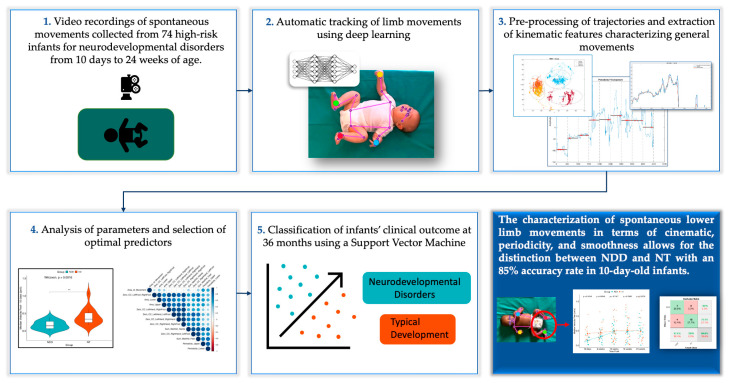
Graphical summary of the model.

Figure 10 provides a detailed graphical summary of the developed model, illustrating its workflow and highlighting its intended applications.

## 4. Discussion

In this study, we employed AI to automatically track infants’ movements and analyze their trajectories to characterize their kinematic behaviors and general movements (GMs). We also investigated how motor skills during the early months of development can predict the onset of NDD in later stages of growth. Our objective was to identify early biomarkers that could differentiate between children who later develop NDD and those who do not, thereby facilitating timely therapeutic intervention for improved outcomes.

To the best of our knowledge, this is one of the first studies to assess motor performance in such young infants using AI techniques to identify early biomarkers of NDD. The method presented in this paper is entirely automatic, requiring no operator intervention. It enables the evaluation of children’s movements, even when they are clothed, and can be applied to videos that may not be perfectly captured. This makes it particularly well suited for integration into applications designed for remote monitoring of children from the comfort of their homes directly by their parents.

Such accessibility could broaden early motor skill assessments, especially in underserved or rural areas where healthcare facilities may be less accessible, thereby reducing disparities in early detection and intervention for NDD. The automatic tracking method eliminates the need for physical markers on the child, which can restrict their natural movements. This non-intrusive approach is essential for studying infants, as it allows for a more ecological observation of their spontaneous motor behaviors without the risk of discomfort or distraction. As a result, this non-contact tracking method preserves the integrity of observed movements while providing a more natural and comfortable environment for the infant’s motor exploration and development.

At the initial time point, precisely 10 days, our analysis revealed a robust correlation between three parameters—‘Median Velocity of Feet’, ‘Area differing from moving average’ of the lower body, and ‘Periodicity’ of the lower body—with the clinical outcome. Using these features, we achieved an accuracy of approximately 85% in discriminating children with NDD versus typically developing (TD) children. Notably, all parameters that showed significant correlations with clinical outcomes pertain to the feet. These findings underscore the diagnostic relevance of focusing on lower limb movements and highlight the need for further research in this direction.

Our study expands on previous work, such as that by Ref. [23], which identified the acceleration of the right foot as the most crucial parameter for the early identification of abnormal motor patterns. We demonstrate that lower limb parameters are critical as early as 10 days of life, a temporal window not thoroughly explored in prior studies. Additionally, our longitudinal analysis revealed that the significant differences observed between the two groups at 10 days gradually diminished over time. By 6 weeks, these differences were less pronounced, and by 12 weeks, they were negligible. This trend suggests that infants who exhibited delays in spontaneous lower limb movements at birth may gradually compensate as weeks progress but eventually manifest NDD. Further investigations are needed to understand the progression and potential mechanisms underlying these compensations. As in our previous investigations, the time window from birth to 6 weeks is particularly suitable for exploring motor performance and predicting NDD risk [17].

The predominance of lower-limb movement differences at such an early stage can be explained by the developmental trajectory of motor skills. Spontaneous lower-limb movements are among the earliest motor patterns to stabilize in neonates, while upper-limb movements related to environmental exploration emerge later as developmental milestones. Studies by Refs. [47,48] have shown that meaningful differences in hand movements predictive of NDD onset typically appear around six months of age.

This finding aligns with the “neurodevelopmental cascade” theory [5,49]. The motor delay identified at 10 days, assessed through the characterization of simple GMs, may have implications for the development of other motor or social domains in later stages. These aspects, however, were not captured by the indices employed in this study, and additional analyses are necessary to explore the potential implications of early motor delay on broader developmental trajectories.

The deep learning-based tracking methodology used in our study exhibited excellent performance when compared to operator-dependent, validated tracking software. Moreover, it has proven to be highly robust in extracting trajectories, even from amateur videos recorded under non-optimal conditions. This robustness underscores the utility of such approaches in real-world settings, enabling parents and caregivers to participate in the assessment process without requiring extensive training or specialized equipment.

The ability to automatically track and analyze infants’ movements from naturalistic video clips represents a significant advancement. This approach paves the way for the development of systems that support families in conducting preliminary assessments of their children’s motor skills and development at home without the need for healthcare facility visits. As widely documented in the literature, early motor skills have a direct correlation with the subsequent development of communication and social abilities, highlighting the potential value of such systems for the widespread early screening of infants for NDDs.

While prior studies have primarily focused on controlled clinical environments, our validation in both structured and unstructured settings demonstrates the broader applicability of this methodology, making it a practical option for remote monitoring. Such systems could also enable large-scale data collection. This would foster new research opportunities and collaborations across clinical and academic institutions, ultimately enhancing the understanding of neurodevelopmental disorders.

A limitation of this study is the relatively small dataset. However, we are actively in the process of collecting additional data and awaiting confirmation of children’s ages at diagnosis, which will allow us to expand the dataset. This limitation arises from challenges such as the rarity of the high-risk population, the need for longitudinal follow-up to confirm diagnoses, and logistical difficulties in accessing participants, as well as the ethical approvals required. Increasing the dataset will improve the generalization capacity of our models and provide a more comprehensive characterization of infants’ motor performance, including additional features such as fluidity, sudden movements, tremors, and rotations—clinically relevant indicators of NDDs. These features are derived from both existing literature and clinical parameters used in neonatal assessments, ensuring a robust approach to feature extraction.

We are also conducting further analyses at time points where significant variables have not yet been identified. Additionally, leveraging the longitudinal nature of our data, we are performing trend analysis of various parameters across the five time points. This study aims to elucidate how motor skills and characteristics evolve during the early weeks of life, facilitating the identification of patterns that differentiate children with NDD from TD children. In line with the neurodevelopmental cascade theory, we hypothesize that early delays in spontaneous movements may progress to impact more complex motor domains and other developmental areas over time. This longitudinal perspective is critical for understanding how early motor performance relates to later developmental outcomes and for paving the way for more targeted early interventions.

## 5. Conclusions

In summary, our paper introduces an AI-based approach for the automatic tracking of infants’ movements using recorded videos in the initial weeks of life across five different time points, spanning from 10 days to 24 weeks. Through the analysis of parameters derived from the trajectories to characterize infants’ movements, we identified a developmental delay in motor skills at the first time point (10 days after birth) for infants later diagnosed with NDD compared to those with typical development. These delays were notably associated with lower body movements, revealing significant distinctions between the two groups. The novel emphasis on lower limb features, identified as key predictors of NDD at such an early stage, underscores the unique contributions of our study. The observation that such differences emerge shortly after birth, diminish during development, and later manifest as delays in other domains underscores the importance of attentiveness to even subtle anomalies in children’s behavioral patterns from birth. This highlights the critical need for systems capable of promptly and effectively supporting both healthcare providers and families in identifying such anomalies. By validating performance in both structured and unstructured settings, our approach demonstrates its versatility, making it suitable for diverse contexts, including home environments. The proposed system stands out for its transformative potential, as it can be implemented using widely accessible tools like smartphones. Offering a low-cost, scalable solution for early assessment, it provides a practical alternative for early detection and intervention. The fully automatic approach described in this paper, requiring only a simple video recording device such as a smartphone, aligns perfectly with these needs.

## Figures and Tables

**Table 1:** Overall dataset with the number of infant videos available for each time point. Subsequently, for analyses aimed at identifying early motor features predictive of clinical outcomes, only data from children who had received a diagnosis (NDD and TD) were included.

Time Point	NDD	TD	No Label	Drop-Out
10 days	11	15	2	4
6 weeks	18	22	6	4
12 weeks	22	25	6	2
18 weeks	14	26	5	2
24 weeks	18	16	6	1

**Table 2:** Descriptive statistics and results of the non-parametric unpaired two-sample Wilcoxon statistical test between the two groups for the three selected variables at 10 days.

Variable	Group	Median	Min	Max	95% Confidence Interval		Unpaired Wilcoxon Test	
					Lower	Upper	U	*p*-Value
Median Velocity of the Feet [px/s]	NDD	0.145	0.0140	0.385	0.0848	0.249	24	0.002
	TD	0.337	0.1641	1.060	0.2742	0.536		
Area differing from moving average (lower body) [px]	NDD	576.866	86.1931	1361.686	368.1953	878.608	29	0.004
	TD	1003.886	589.6773	1798.358	864.5623	1253.916		
Periodicity (lower body) [1/px]	NDD	0.104	0.0323	0.149	0.0776	0.123	31	0.006
	TD	0.144	0.1020	0.237	0.1261	0.167		

**Table 3:** Post hoc test of the mixed-effects models.

	Comparison								
Variable	Time Point	Group	Time Point	Group	Difference	SE	t	Df	*p*
Median Velocity of Feet	10 days	NDD	10 days	TD	−0.25532	0.1262	−2.0234	177	0.045
	6 weeks	NDD	6 weeks	TD	−0.14762	0.1019	−1.4484	173	0.149
	12 weeks	NDD	12 weeks	TD	−0.02627	0.0942	−0.2790	169	0.781
	18 weeks	NDD	18 weeks	TD	−0.07217	0.1061	−0.6801	175	0.497
	24 weeks	NDD	24 weeks	TD	0.00852	0.1098	0.0776	176	0.938
Area differing from moving average (lower body)	10 days	NDD	10 days	TD	−488.51	232	−2.1036	177	0.037
	6 weeks	NDD	6 weeks	TD	−329.69	190	−1.7363	167	0.084
	12 weeks	NDD	12 weeks	TD	−51.00	177	−0.2888	158	0.773
	18 weeks	NDD	18 weeks	TD	−240.54	197	−1.2201	170	0.224
	24 weeks	NDD	24 weeks	TD	−89.90	203	−0.4418	173	0.659
Periodicity (lower body)	10 days	NDD	10 days	TD	−0.04338	0.0260	−1.6703	177	0.097
	6 weeks	NDD	6 weeks	TD	−0.01417	0.0211	−0.6709	170	0.503
	12 weeks	NDD	12 weeks	TD	−0.00121	0.0196	−0.0616	163	0.951
	18 weeks	NDD	18 weeks	TD	−0.01011	0.0219	−0.4605	173	0.646
	24 weeks	NDD	24 weeks	TD	0.01251	0.0227	0.5515	174	0.582

**Table 4:** Performance metrics achieved for the first time point (10 days) by the SVC classifier.

	Accuracy	Precision	Sensitivity	F1 Score	Specificity
10 days	84.62%	100%	63.64%	77.78%	100%

## Data Availability

The dataset presented in this study is available on request from the corresponding author due to ethical restrictions.

## Supplementary Materials

The following supporting information can be downloaded at: https://www.mdpi.com/article/10.3390/diagnostics15020136/s1, Table S1: Comprehensive dataset overview providing details on the videos available for each participant, along with their corresponding labels.
