# Supplementary material for: Marker-Less Video Analysis of Infant Movements for Early Identification of Neurodevelopmental Disorders
Source: Diagnostics (Basel). 2025 Jan 8;15(2):136. doi: 10.3390/diagnostics15020136 (PMC11763807; doi:10.3390/diagnostics15020136)
Supplement: Supplementary file 1 [file diagnostics-15-00136-s001.zip › diagnostics-3378852-supplementary.pdf]

*Table S1. Comprehensive Dataset Overview providing details on the videos available for each participant, along with their corresponding labels.*

| Subject | 10 days | 6 weeks | 12 weeks | 18 weeks | 24 weeks | Label |
|---|---|---|---|---|---|---|
| LO0002 | | X | | | X | NDD |
| LO0015 | | | X | | X | NDD |
| RM0001 | | X | X | X | X | NDD |
| RM0002 | X | X | X | | X | NDD |
| RM0003 | | X | X | X | | NDD |
| RM0006 | X | X | X | X | X | NDD |
| RM0007 | X | X | X | X | X | TD |
| RM0008 | X | X | | X | X | NDD |
| RM0011 | | X | X | X | | TD |
| RM0012 | | X | | | | TD |
| RM0013 | X | | X | X | X | TD |
| RM0014 | | X | X | X | X | TD |
| RM0018 | X | X | X | X | X | Drop-out |
| RM0019 | X | X | | | | Drop-out |
| RM0020 | X | X | X | X | X | TD |
| RM0021 | | | | | X | No Label |
| RM0022 | | X | X | X | X | TD |
| RM0023 | | X | X | | | NDD |
| RM0024 | X | | X | | X | NDD |
| RM0025 | X | X | X | X | | TD |
| RM0028 | | | X | X | X | TD |
| RM0029 | | X | X | | X | NDD |
| RM0030 | X | X | X | X | | TD |
| RM0031 | | | | | X | NDD |
| RM0032 | | X | X | X | X | TD |
| RM0033 | | | X | | | NDD |
| RM0034 | X | | X | | | NDD |
| RM0036 | X | | X | X | | TD |
| RM0037 | | X | X | X | X | TD |
| RM0038 | X | X | X | X | X | TD |
| RM0040 | X | | X | X | X | NDD |
| RM0041 | | | X | X | X | TD |
| RM0042 | | X | X | X | | NDD |
| RM0043 | | | | | X | NDD |
| RM0044 | X | X | X | | | TD |
| RM0048 | X | X | | | | Drop-out |
| RM0050 | | | | X | | TD |
| RM0054 | | | X | X | | TD |
| RM0055 | | | X | | | Drop-out |
| RM0057 | | X | | | | TD |
| RM0059 | | X | X | | X | NDD |
| RM0066 | X | | | | | NDD |
| RM0069 | X | X | | | X | TD |
| RM0073 | X | X | | X | X | TD |

| | | | | | | |
|---|---|---|---|---|---|---|
| RM0074 | | X | X | | X | TD |
| RM0075 | X | X | X | X | X | TD |
| RM0077 | | | | X | | TD |
| RM0078 | | | X | X | X | TD |
| RM0079 | X | | X | X | | TD |
| RM0080 | | X | X | X | | TD |
| RM0083 | X | X | | X | | Drop-out |
| RM0086 | | X | X | X | | TD |
| RM0087 | X | X | X | | | TD |
| RM0088 | X | X | X | | | No Label |
| RM0091 | X | X | X | X | X | NDD |
| RM0092 | X | X | X | X | | TD |
| RM0094 | | | | X | | TD |
| RM0095 | | X | | | | TD |
| RM0096 | X | X | X | X | X | No Label |
| RM0097 | | | X | X | | NDD |
| RM0101 | | X | X | X | X | No Label |
| RM0102 | X | | X | X | X | TD |
| RM0103 | | | X | X | X | NDD |
| RM0104 | | X | X | X | X | NDD |
| RM0105 | | X | X | X | X | No Label |
| RM0107 | | X | X | X | X | NDD |
| RM0108 | | X | X | X | | No Label |
| RM0113 | | | | X | X | No Label |
| RM0114 | X | X | X | | | NDD |
| RM0115 | | X | X | | X | No Label |
| RM0119 | | X | | | | NDD |
| RM0121 | | X | X | X | | NDD |
| RM0122 | X | X | X | X | X | NDD |
| RM0125 | X | X | X | X | X | NDD |
